# The Role of Oxytocin and the Effect of Stress During Childbirth: Neurobiological Basics and Implications for Mother and Child

**DOI:** 10.3389/fendo.2021.742236

**Published:** 2021-10-27

**Authors:** Michael H. Walter, Harald Abele, Claudia F. Plappert

**Affiliations:** ^1^ Department of Midwifery Science, Institute for Health Sciences, University Hospital Tübingen, Tübingen, Germany; ^2^ Department for Animal Physiology, Institute of Neurobiology, University of Tübingen, Tübingen, Germany; ^3^ Department for Women’s Health, University Hospital Tübingen, Tübingen, Germany

**Keywords:** breastfeeding, midwifery, mother–infant bonding, neuroendocrinology, pregnancy

## Abstract

The neuropeptide oxytocin acts as a hormone and a neuromodulator, influencing a multitude of human social behaviors, including reproduction. During childbirth and the postpartum period, it plays a key role in regulating and controlling processes that ensure a safe birth and the health of mother and child. Especially the onset of labor, the progress of labor and initial breastfeeding are mediated by oxytocin. In the maternal brain it controls the initiation of the mother–infant bond and the mother’s emotional responses towards her child. In this review we summarize the current state of knowledge about the role of oxytocin during the different aspects and mechanisms of human childbirth, combining research from human and animal studies. Physiological and psychological stress during childbirth and lactation can have negative effects on the progress of labor, breastfeeding and bonding. We discuss how maternity caregivers can support the positive effects of oxytocin and minimize the effects of stress. Furthermore, we highlight aspects of the basic neurobiological principles and connections where further research is needed to improve our understanding of the regulation and the effects of oxytocin to support maternal and infant health.

## Introduction

Childbirth, the early phase of the postpartum period, and lactation are regulated by neuroendocrine processes, which act in a neurochemical cascade to facilitate the physiological progress of giving birth and the transition to motherhood (1). The peptide hormone oxytocin plays a crucial role in this process and is therefore of utmost importance for all professionals involved in maternal caregiving (2), especially for midwives who carry a large share of the responsibility for the health of mothers and their children during physiological birth (3). Stress and the consequent release of hormones, e.g., cortisol, has been shown to be a major factor affecting all aspects of childbirth, lactation, and the development of the mother–infant bond, yet the direct connection of these behavioral observations with their hormonal basics is mostly unknown. In this review article we describe the major neurobiological principles and theories relevant to the production and release of oxytocin, drawing evidence from animal and human studies, and how it acts as a hormone and as a neuromodulator during childbirth and the postpartum period. We aim to highlight where further research is needed to understand the exact molecular mechanisms in which oxytocin and other hormones act during childbirth and the postpartum period and where and how clinical manipulation of oxytocin levels is indicated.

## Production and Primary Sources of Oxytocin in Mammals

**Figure 1:**
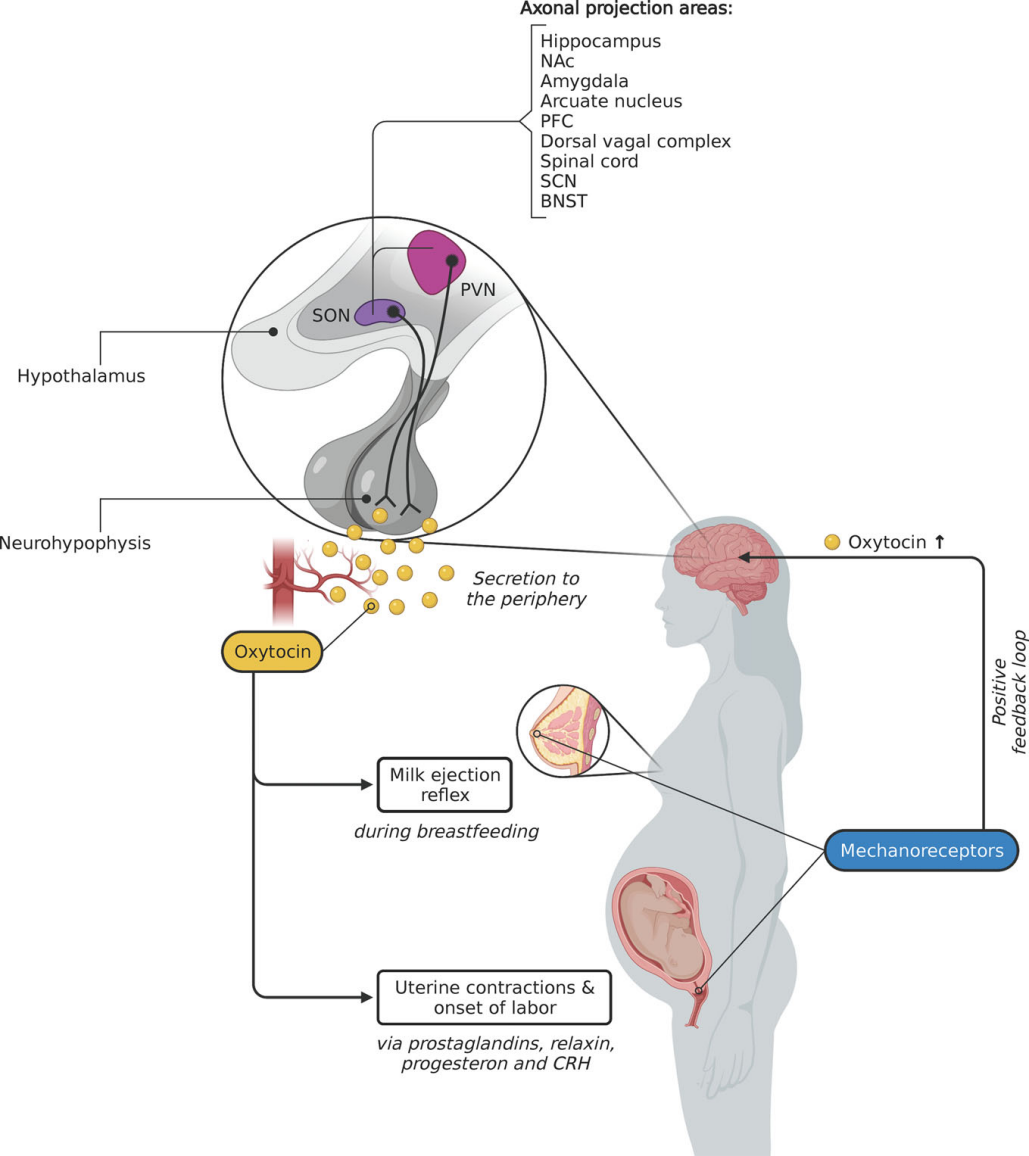
Peripheral effects of central oxytocin release during childbirth and lactation. The neuropeptide oxytocin is mainly produced by magnocellular neurons in the paraventricular nucleus (PVN) and the supraoptic nucleus (SON) of the hypothalamus. Axons of these neurons terminate in the neurohypophysis, where oxytocin is secreted – in pulses – into the blood. Mechanoreceptors in the nipple (activated by the suckling newborn) and the cervix (cf. Ferguson reflex) create a positive sensory feedback loop, which leads to the additional release of oxytocin in the brain. Oxytocinergic neurons in the hypothalamus also release oxytocin to other brain areas (sources: 7–9) *via* axonal transport and *via* dendritic release (not shown). NAc, Nucleus accumbens; PFC, Prefrontal cortex; SCN, Suprachiasmatic nucleus; BNST, Bed nucleus of the stria terminalis. Created with BioRender.com.

Knowledge about sources and transport mechanisms of oxytocin in mammals mostly comes from research on rodents and other animal models, and most mechanisms have been shown to be evolutionary conserved and presumably also apply to all other mammals (4). Oxytocin is produced by magnocellular neurosecretory cells within the paraventricular nucleus (PVN) and the supraoptic nucleus (SON) in the hypothalamus (5–7). After synthesis it is transported along the axons of these neurons to the neurohypophysis where it is secreted into the bloodstream in pulses (**Figure 1**). In addition to this global release mechanism, oxytocin is produced locally in specialized cells of the uterus, amnion, chorion and decidua, where it acts as a paracrine signal to influence the behavior of neighboring cells (10). Oxytocin also acts as a neuromodulator, altering the activity of other neurons in the central nervous system (CNS) of mammals. Parvocellular, oxytocinergic neurons in the PVN of mice project to other brain areas, including the prefrontal cortex and basal areas of the limbic system, i.e., the hippocampus, amygdala and nucleus accumbens (5, 11, 12). These brain areas widely express the oxytocin receptor (OXTR; 13) and its expression density increases shortly before birth, caused by the increase of the ratio of estrogen/progesterone, enabling these regions to be modulated by oxytocin (4, 14). The brain regions involved are part of a network that is associated with reward, sociosexual behavior, memory formation, and the regulation of emotions (**Figure 1**) (13). In addition to axonal transport mechanisms, the release of oxytocin is also mediated by dendrites of neurons in the SON and PVN, leading to a flooding of close-by and further away brain areas (7). Degradation of oxytocin after binding to its receptor in the CNS is much slower than the degradation of oxytocin in the bloodstream, which results in persistent behavioral effects (7). The concentration of oxytocin in the CNS is not correlated with blood concentration since peripheral oxytocin is unable to pass the blood–brain barrier, which makes a direct effect of peripheral oxytocin on the CNS unlikely. Therefore, any conclusion based on a correlation between peripheral and central oxytocin levels has to be taken with caution (13, 15).

## The Role of Oxytocin at the Onset of Human Labor

In humans, premature babies, born 2–6 weeks before the expected date of birth, carry an increased risk for lifelong health problems (16) and infants born after a postterm pregnancy have a higher risk for perinatal morbidity and mortality (17). To give the newborn the greatest chance of survival a series of finely tuned mechanisms have been selected for during the course of human evolution to initiate the beginning of childbirth at the right point in time. These include the switch of uterine muscle activity from a resting state, characterized by single, unsynchronized contractions during pregnancy, to a state of coordinated uterine contractions at the point in time when the fetus is mature. In addition, the cervix has to ripen and efface before it can dilate in order to allow the descent of the fetus from the uterus to the vagina. Both of these mechanisms are triggered by processes days or weeks before the actual onset of labor (18) and happen in the fetomaternal region, an area of interaction between mother and fetus, and in the decidua, placenta and the chorioamnion (19). The exact mechanisms of these processes are still under investigation (18, 20), including the question if they are initiated by the mother (in the myometrium or placenta) or by the fetus *via* the hypothalamic–pituitary–adrenal (HPA) axis. Many hormones, including estrogen, progesterone, prostaglandins, corticotropin-releasing hormone (CRH), relaxin, and oxytocin act in parallel and interactive signaling pathways to initiate the onset of labor (18, 21). These hormones combine an endocrine function, being secreted into the blood and reaching their destination *via* the bloodstream and exert a paracrine function, acting locally on neighboring cells (21, 22). Certain processes have been shown to progress complementarily thereby enabling the compensation of one another in case one factor shows loss of function (23), indicated for example by the finding that genetically modified knockout mice not expressing the oxytocin receptor are still able to bring forth pups (24).

Oxytocin has been described as the key player in these processes, yet evidence for its direct role remains inconclusive or absent since direct measurements of blood plasma oxytocin levels are seldom taken in the context of human reproductive behavior [cf. (25) for a review about measurements of peripheral oxytocin levels]. The pulsatory secretion in the neurohypophysis in combination with the low half-value period of blood oxytocin requires an adequate sampling rate when measuring its blood concentration. Sampling rates in previously conducted research were highly variable, making conclusions difficult (26).

Vannuccini et al. (18) have postulated that oxytocin plays an important but not critical role at the onset of childbirth. Nonetheless, it has been shown that the number of uterine oxytocin receptors increases up to 200-fold towards the end of gestation (5, 27), caused by the increase of the estrogen/progesterone ratio, which neutralizes the progesterone-mediated inhibition of OXTR production in the myometrium. The density of prostaglandin receptors increases alongside the OXTR density as well as the synthesis of enzymes, which are responsible for the contraction of the myometrium (28).

At the onset of labor, estrogen synthesized in the placenta stimulates the local synthesis of oxytocin in the amnion, chorion and decidua (26), evident by the presence of oxytocin mRNA (10). This local synthesis is independent of the endocrine secretion in the hypothalamus, hence explaining why oxytocin has been detected locally in cells but not in blood samples (29) and why no increase in blood plasma concentration of oxytocin during pregnancy and the beginning of childbirth in women has been reported (30). This paracrine produced oxytocin in the amnion acts *via* a direct and an indirect mechanism to mediate uterine contractions. Indirectly, it stimulates the synthesis of prostaglandins E_2_ and F_2α_, which in turn trigger uterine contractions, leading to an increase in OXTR density and contributing to the formation of gap junctions between smooth muscle cells of the uterus (18). Directly, it activates Ca^2+^ channels in the smooth muscle cells, resulting in a release of Ca^2+^ from the sarcoplasmatic reticulum that initiates the muscle contraction cycle (20).

Before uterine contractions begin, an inflammation-like process in the amnion and chorion, characterized by an increase in cytokines, chemokines, as well as prostaglandins E_2_ and F_2α_, has been observed. These processes cause biochemical changes of fetal membranes and the ripening of the cervix, initiating parturition (31). Inflammatory activation is also caused by direct action of oxytocin on the release of cytokines (32).

## The Role of Oxytocin During Childbirth and the Involution of the Uterus

During human parturition, the measurable amount of blood plasma oxytocin increases: it doubles during the latent phase of dilatation and increases further until the second stage of labor (33). The pulsatile secretion of oxytocin by the neurohypophysis increases in amplitude and frequency during childbirth, reaching a maximum of three pulses within ten minutes shortly before delivery (34). These oxytocin pulses are triggered by signals within the CNS and by the pressure that is exerted by the fetus on mechanoreceptors of the cervix and the vaginal walls *via* a positive feedback loop, termed Ferguson reflex. From research in rodents we know that intense, rhythmically appearing neural signals have been recorded in the PVN and SON in the hypothalamus, which receive their input from the sensory neurons in the cervix and vagina (35). This rhythmic activity in the hypothalamus leads to the pulsatory release of oxytocin in the brain and into the bloodstream (**Figure 1**) (36, 37). When the exerted pressure on the cervix by the fetus reaches a maximum, that is during the fetal expulsion stage and shortly after birth, the oxytocin concentration increases 3- to 4-fold compared to the onset of labor (33, 34). In rats it was shown that a hypothalamic opioid-dependent neural circuit prevents an overshoot of oxytocin levels (38). A study by Goodfellow et al. (39) found that in women receiving epidural analgesia, blood oxytocin concentration is reduced, presumably because the administered anesthetics and opioids prevent the transmission of afferent neural signals. It is currently being discussed that this is the reason why the longer the epidural analgesia is administered, the higher the rate of non-spontaneous births and the use of synthetic oxytocin (40). Medical intervention during birth by means of emergency caesarian section and/or epidural analgesia also adversely affect the initiation of breastfeeding after birth by reducing oxytocin and prolactin levels (41).

Surprisingly, the pulsatile release profile of oxytocin and the frequency of uterine contractions are not temporally correlated (42). This can be because contractions of the uterine muscle cells are additionally controlled by the parasympathetic nervous system overlaying the frequency of the oxytocin pulses. Projections from the PVN to parasympathetic nerves in the lumbosacral region of the spine, activated by central oxytocin, cause uterine contractions and additionally improve circulation in the uterine muscles, at least in rats (43, 44).

For the involution of the human uterus, i.e., the reduction of the uterus to prepartum size and condition after birth, tonal contractions of the uterine muscles are essential. These specialized contractions are also mediated by oxytocin (18). The highest levels of peripheral oxytocin have been measured 15 minutes after delivery (45). These high postpartum levels are achieved by activation of the hypothalamus, induced by the skin contact of mother and child and the stimulation of the mother’s nipples through breastfeeding (46–48).

## The Role of Oxytocin During Lactation

A number of studies in rodents have revealed that oxytocin also plays a pivotal role during lactation. Wagner and colleagues (49) found that mammary glandular tissue of female OXTR-knockout mice is reduced within twelve hours after giving birth, despite the pups’ suckling. When a pup suckles its mother’s breast, mechanosensitive receptors are activated in the areolar region. These neurons are oxytocinergic and project *via* the spinothalamic tract to the hypothalamus, where they trigger the activation of oxytocin-producing neurons leading to a pulsatory secretion of oxytocin into the bloodstream (13, 50). A positive feedback loop within the SON in which oxytocin acts on its own release leads to an amplification of the amount of oxytocin that is secreted (**Figure 1**) (6). This mechanism ensures that the necessary amount of oxytocin in the mammary glands is available (51). Secretion by these neurons in the SON occurs in bursts every 5–15 minutes and lasts for 3–4 seconds (5). In the mammary glands, blood plasma oxytocin binds to its receptor expressed in the myoepithelial cells of the walls of the lactiferous ducts and in the epithelial cells of the alveolar glands. Oxytocin causes contractions in the myoepithelial cells, which increases the pressure in the breast, resulting in a wavelike release of milk from the mammary glands through the lactiferous ducts (5, 13). This milk ejection reflex, or let-down reflex, appears 30–60 seconds after the infant has begun suckling and is viable during the whole time a mother breastfeeds her child (5).

The number of oxytocin receptors in these myoepithelial cells in rats is upregulated during gestation in a similar manner as in the myometrium (52). A study by Erickson and Emeis (53) has shown that the administration of exogenous oxytocin during human childbirth can have negative consequences for breastfeeding. We suspect that this is because synthetic oxytocin causes the internalization of oxytocin receptors hence resulting in lower activation levels by oxytocin which are required for successful lactation (5).

The milk ejection reflex is not only activated by the infants’ suckling and, hence, the activation of peripheral neurons in the SON, but also by the activation of central oxytocinergic neurons. These are triggered by other external stimuli, e.g., the infant crying which elicits a dendritic release of central oxytocin (54) and a central activation of the milk ejection reflex, even before the infant begins to suckle. This external trigger mechanism of the milk ejection reflex has been shown to be an important factor for successful, long-lasting breastfeeding (5).

## The Role of Oxytocin in Mother–Infant Bonding

The simultaneous activity of the neuropeptide oxytocin during childbirth, both in the periphery and in the brain, is a fascinating example of the result of evolutionary processes that ensure a species’ successful reproduction and thus its survival. In the brain, oxytocin acts as a neuromodulator in multiple neural circuits *via* axosynaptic and dendritic projection from the SON and PVN, which are essential for the control of reproductive behaviors (55). Oxytocin also contributes to other behaviors such as food intake, learning and memory, and addiction to opioids (5). Oxytocin has additionally been associated with promoting human social interactions (56) and has been shown to reduce fear and pain, as well as physiological and psychological stress (34, 57). Maternal care behaviors, the development of the mother–infant bond and maternal aggression to protect the own young are directly influenced and facilitated by oxytocin (13, 58). Most of what we know about the role of oxytocin in maternal behaviors comes from animal studies, mainly from findings in rodents and sheep, e.g., OXTR-deficient mice show higher levels of pup abandonment compared to wildtype controls (59). In all mammals, mother and young have to go through two steps to form their bond: first, a selective recognition process that has to happen in a short period of time after birth and, second, the establishment of a permanent affection and attraction (6, 60). The first step, the recognition process, is mediated under hormonal, including oxytocinergic, control (61), whereas the process to implement a long-lasting connection is based on structural changes in the mother’s brain and becomes independent of hormonal control (62, 63). Research in rodents has revealed that the initiation of the mother–infant bond by recognition of the young is regulated by an interaction between oxytocin, estrogen, and prolactin in the anterior hypothalamus and the stria terminalis, which connects the hypothalamus with the amygdala (61). Furthermore, these nuclei interact with the dopaminergic reward system, which in turn controls maternal motivation. Numan and Young (61) also found that projections from the olfactory system (olfactory recognition of the young) and the amygdala (responsible for the evaluation of emotional valence) are crucial for the initiation of the bond. The oxytocin required for these processes stems from oxytocinergic projections from the PVN to the anterior hypothalamus, which are activated by the Ferguson reflex and nipple stimulation (62).

Little is known about the effect of central oxytocin on bonding behavior of human and non-human primates. A direct manipulation of central oxytocin can be achieved by intracerebral injection into the ventricular system in various animal models, like sheep and rodents, activating the onset of maternal behaviors and facilitating the bonding process (64, 65). In humans, a successful method to influence central levels is to administer oxytocin intranasally. After administration the activation of brain regions can be measured, e.g., using fMRI to determine changes in the activation of brain regions and in the behavioral response to exogenous oxytocin (66). Peripheral oxytocin levels are also under investigation in bonding research. Feldman et al. (67) found that plasma oxytocin levels were stable across pregnancy and the postpartum period and related to the emergence of a set of maternal bonding behaviors. A recent study investigated the effects of maternal behavior of mothers with infants aged 4–24 months and found that synchronous maternal behavior (an indicator for high quality maternal care) was associated with increased dopamine responses, stronger intrinsic connectivity within the medial amygdala network and a decrease in plasma oxytocin (68). Further research is required to expand our understanding of the role of central and peripheral oxytocin in human mother–infant, and potentially father–infant, bonding (69). To carry out this research, researchers should take into account recent findings that suggest that oxytocin is present in different functional states in human blood plasma samples and that it potentially exerts its effects after degrading into various active fragments (70). Commercially available methods have been shown to detect these different states of human blood plasma oxytocin with variable specificity (71), likely explaining the high variance of human blood plasma oxytocin concentrations reported in the literature (72).

Contrary to the adult brain, the blood–brain barrier of the fetus is permeable for peripheral oxytocin from its mother’s circulatory system. In rodents it was shown that a systemic administration of oxytocin to the dam during birth has a long-term impact on the behavior of the pups (73). Later in life, these pups exhibited an improvement in caregiving to their own pups and an increase in the number of social interactions with each other in their adult life stage. Epigenetic changes in the gene encoding for the OXTR in the fetal brain (increased DNA methylation at the OXTR promotor) and an increase in the total number of oxytocin receptors are discussed as potential explanations for these findings (73). It remains to be investigated if maternally given oxytocin during labor, childbirth, or after giving birth have an (epigenetic) effect on the child’s oxytocin system in humans (74).

## The Effects of Stress on the Oxytocin System

Anxiety has been shown to prolong the time to give birth and this is correlated to low blood plasma concentrations of oxytocin in women (75). Additionally, Thomas et al. (76) found a positive relationship between the length of parturition and the concentration of β-endorphin, an endogenous opioid that is released during time of stress. It is being discussed if the prolongation of parturition under stress is caused by an opioid-dependent reduction of oxytocin release, as this was shown in rats (77). This is achieved by two means: first, opioids inhibit the neurosecretory terminals in the neurohypophysis *via* binding to κ-opioid receptors (78) and, second, by reducing the pulse rate of oxytocinergic neurons of the PVN *via* binding to μ-opioid receptors (79). Support for these proposed mechanisms comes from further studies in rodents in which it was shown that oxytocin infusions and the administration of the opioid antagonist naloxone can mitigate the prolongation of parturition caused by disturbing the dam (77). The inhibition and regulation of oxytocin secretion through the effects of opioids serves to control the contractions during childbirth and to prevent uterine tachysystole. After birth, the number of opioid receptors and the concentration of β-endorphin in the hypothalamus is reduced. This builds the basis upon which the extremely high postpartum oxytocin concentrations are achieved (79). From these findings it is likely to conclude that stress, which is caused by disturbance of the mother during childbirth, leads to an increase in the opioid-mediated inhibition of oxytocin secretion and thereby to a reduction in uterine contractions that will have a negative effect on the progress of labor.

A second mechanism that has been identified as a factor in the slowing of labor under stress is mediated by the autonomic nervous system. Oxytocin is known to activate parasympathetic projections in rats, leading to an increased blood flow into the uterine muscles and a widening of uterine arteries (43), ensuring the fetal oxygen supply even during uterine contractions in cows and horses (80). Therefore, oxytocin causes a shift in activity of the autonomic nervous system from the sympathetic to the parasympathetic nervous system (81). This change is measurable, for example by the heart rate variability (HRV), which is greater under parasympathetic control. Stressful situations during birth alter the autonomic nervous system by increasing the dominance of the sympathetic over the parasympathetic nervous system (i.e., lower HRV) by activating β_2_ adrenoreceptors through adrenalin and noradrenalin (80, 82). There is evidence in pigs that the activation of these receptors causes an inhibition of uterine contractions and therefore a slowing of labor (83). An effect of stress on the release of oxytocin has been shown in pigs (83) but not in horses (82), probably due to methodological differences, e.g., different sampling rates in measuring oxytocin concentrations.

The pulsatory stress caused by the rhythmic contractions of the uterus during labor causes a tend-and-befriend reaction of the mother, contrary to the usual fight-or-flight response to stress mediated by the sympathetic nervous system. The biological basis of this tend-and-befriend reaction, first described by Taylor et al. (84), appears to be oxytocin and its interplay with estrogen, which ensures the safety of delivery and the appropriate behavior of the mother after birth (46). Corroborating this hypothesis, previous studies have found that oxytocin has an anxiolytic effect (34, 85, 86), and is able to regulate the stress response *via* oxytocinergic projections which connect the hypothalamus with the hippocampus, amygdala and prefrontal cortex (87). Exogenous stress during labor leads to a dominance of the sympathetic nervous system, a shift in response from tend-and-befriend towards fight-or-flight and the release of catecholamines, which can slow labor progress (88).

Acute stress also has negative effects on lactation. If the sympathetic nervous system is highly active, it has an inhibiting effect on the hypothalamus and, hence, the pituitary gland. This causes a reduction in the release of oxytocin and prolactin. Furthermore, it causes a local vasoconstriction of the nipple and an overactivity of the myoepithelial cells in the mammary gland. These factors contribute to a disruption of milk production and the milk ejection reflex (89). This is supported by a recent systematic review by Uvnäs-Moberg et al. (41), which shows that stress reduces the number of oxytocin pulses during early breastfeeding.

It should also be noted that the mother’s behavior itself is affected by an increase in stress. Animal research in rodents has shown that intracranial injections of CRH into the ventricular system inhibit certain components of maternal behavior (64, 90). Findings about the role of CRH on maternal behavior are supported by observations of human patients who have suffered from early childhood trauma. These patients showed a chronic overactivation of the CRH system as well as the HPA axis and impairment of parental behavior (64). In non-human primates, mothers who received injections of CRH into their ventricular system spend less time with their offspring (64). It is likely that changes in the oxytocin system are the underlying cause for the described effects on the mother–infant bond, which remains to be studied.

## Clinical Consequences of Oxytocin Manipulation

The crucial task for all maternity caregivers is to support the mother’s innate biological processes and to carefully balance the benefits and dangers of any intervention. Further research is required to estimate the potential effects of a prolonged infusion of synthetic oxytocin on its natural pulsatory release profile, which is essential for a normal birth process. Two aspects should be considered when administering synthetic oxytocin during childbirth. First, the dosage of synthetic oxytocin should not exceed the physiological blood plasma concentration of 9 mU/min (34, 91). A recent study by Daly and colleagues (92) has shown that the dosage of synthetic oxytocin during the onset of labor and the subsequent birth varies substantially between clinics and countries. In Germany, dosages up to 27 IU within eight hours have been documented. Secondly, a constant infusion of synthetic oxytocin results in a flattening of the natural oxytocin pulses (34). An infusion with synthetic oxytocin, mimicking the naturally occurring pulses, could potentially maintain the amplitude and frequency of uterine contractions and prevent an overstimulation. This could also slow down the downregulation of OXTR density (13, 34). Gimpl and Fahrenholz (5) have shown that in fibroblast cells more than 60% of all oxytocin receptors are internalized within 5–10 minutes after receptor stimulation, thereby drastically reducing the binding capacity of these cells for oxytocin.

It is also unknown how epidural analgesia and the associated reduction (or the complete suppression) of the Ferguson reflex, which ensures the sufficient oxytocin concentration during and after birth (see above), affects the physiological processes of mother and child. Among the many other positive aspects that are mediated by oxytocin, an elevated oxytocin concentration after birth contributes to the emergence of the mother’s positive emotions towards her baby and towards herself (46, 88). Maternal satisfaction with birth is achieved by an interaction of oxytocin with the dopaminergic reward system (93, 94). Oxytocin also has an amnestic effect and lets the mother forget about the painful aspects of labor and childbirth (34, 95). Maintaining a normal level of oxytocin during childbirth and afterwards has therefore not only an effect on the way in which the mother experiences the birth of her child but also on her own mental well-being (96).

Surprisingly little is known about how midwives can support mothers during these processes and how oxytocin levels can be increased (or maintained) physiologically, although this should be one primary focus of midwifery work (3). For example it has been shown in a study by Lund et al. (97) that rhythmic, massage-like stimulation of the skin increases the blood plasma oxytocin concentration and reduces the amount of nociception. It is known that stimulation of the breast before parturition also leads to an increase in blood plasma oxytocin levels (98). However, it remains to be investigated if these measures can have a positive effect on the progress of labor. Likewise, studies about the influence of body position during birth on the oxytocin system are missing (99). More research is needed to inform guidelines on supporting women in labor, based on scientific evidence about promoting the natural release of oxytocin and the effects of synthetic oxytocin administration during birth (100).

We postulate that the professional support of women in the postpartum period should always have the reduction of stress-causing disturbances from intrinsic and extrinsic factors and the support of the mother–child bond (including breastfeeding) as its primary goals. Important factors that have been shown to reduce stress during birth are the birth environment, which can hinder or support physiological birth depending on the stress level exerted on the birthing woman (101), as well as intrapartum care with minimal intervention and birth preparedness (102), which requires a one-to-one support during labor and birth by birth attendants, mainly midwives. It has been shown that this social support reduces labor stress and pain (1).

Continuous support by a midwife has also been shown to have a positive effect on the mother’s self-determination and self-confidence (103). Further studies should explore the mediating effect on stress of the midwifery model of care, including continuity of care during the entire childbearing trajectory and one-to-one support during labor (104) and its potential impact on the oxytocin system.

## Conclusions

The neuropeptide oxytocin plays a central role in securing the health and safety of mother and child during birth and beyond. It acts by endocrine and paracrine mechanisms, both in the periphery and as a neuromodulator in the central nervous system. The processes in which oxytocin is released, binds to its receptor, and affects various aspects of childbirth are finely tuned and strictly regulated, both temporally and spatially. Additionally, oxytocin influences a large bandwidth of basic biological functions of human social behavior, including recognition, trust and empathy (105). By lying the basis for reproductive pair-bonds it ensures our species’ survival. It does so by directly supporting childbirth and lactation and by affecting the emotional processes of parental care, pair bonding and social interactions by changes in the physiology and anatomy of maternal brains. Considering that maternal oxytocin levels during childbirth can have an epigenetic effect on the infant’s brain, everyone involved, clinicians, midwives, and mother and father carry a great responsibility for the well-being of the mother and the health of the next generation (73, 106). It is therefore vital to increase our understanding of the role of oxytocin and how its release can and should be influenced. A deeper knowledge of the underlying processes will influence and improve future obstetrics and mainly the work of midwifes who are important attendants and confidents of women before, during and after birth (107) to ensure the health of mothers and a safe start in life for their children.

## Author Contributions

CP wrote the first draft of the manuscript. MW, HA, and CP edited the manuscript. All authors contributed to the article and approved the submitted version.

## Conflict of Interest

The authors declare that the research was conducted in the absence of any commercial or financial relationships that could be construed as a potential conflict of interest.

## Publisher’s Note

All claims expressed in this article are solely those of the authors and do not necessarily represent those of their affiliated organizations, or those of the publisher, the editors and the reviewers. Any product that may be evaluated in this article, or claim that may be made by its manufacturer, is not guaranteed or endorsed by the publisher.
